# Defining and Detecting Crossover-Interference Mutants in Yeast

**DOI:** 10.1371/journal.pone.0038476

**Published:** 2012-06-06

**Authors:** Frank Stahl

**Affiliations:** Institute of Molecular Biology, University of Oregon, Eugene, Oregon, United States of America; National Cancer Institute, United States OF America

## Abstract

The analysis of crossover interference in many creatures is complicated by the presence of two kinds of crossovers, interfering and noninterfering. In such creatures, the values of the traditional indicators of interference are subject not only to the strength of interference but also to the relative frequencies of crossing over contributed by the two kinds. We formalize the relationship among these variables and illustrate the possibilities and limitations of classical interference analysis with meiotic tetrad data from wild-type *Saccharomyces cerevisiae* and from *mlh1* and *ndj1* mutants.

## Introduction

The tractability of yeast for studies of meiosis has encouraged the search for mutants that might illuminate an elusive classical linkage phenomenon, crossover interference. An impediment to such a search is posed by the presence of two kinds of crossing over, with and without interference. Mutants that reduce interference as measured by the coefficient of coincidence [1] or the nonparental ditype (NPD) ratio ([2], [3]), might do so by reducing interference between the interfering crossovers, by reducing their frequency, by increasing the frequency of noninterfering crossovers, or by introducing population heterogeneity as a result of faulty effective pairing [4] or by combinations of these factors. The relationship between linkage map distance and crossover interference in creatures with two kinds of crossovers has been the subject of several statistical analyses (*e.g*. [5]–[11]). All were based on the assumption that the noninterfering crossovers are sprinkled along the chromosomes independently of each other and of the interfering crossovers. Typically, they further assumed that interfering crossovers are distributed with respect to each other according to an Erlang or Gamma distribution. These assumptions have usefully described the distribution of crossovers along the linkage maps in wild-type backgrounds (reviewed in [9]). They have estimated the numbers of crossovers of each kind as well as the values of indices (*m* or *gamma*) of interference for the interfering crossovers, which reflect the degree of modality in the frequency distribution of intercrossover distances. Such analyses, however, require many markers, a computer program of rather daunting nature, and an assumption that the relative contributions of the two kinds of crossovers are constant along the chromosome. Here I derive a model-independent alternative – a simple formula that exposes the relationship between classical indicators of crossover interference, on the one hand, and the contributions of each of the two kinds of crossovers on the other. The formula has the advantage of being applicable to analyses of crossing over and interference in single short intervals or in pairs of short, adjacent intervals. One shortcoming of the method is that it requires data from more tetrads than do the methods referenced above. Another is that the confounding of variables prevents assumption-free estimates of their values.

## Results

### Basic Algebra for Two Pathways

We describe the genetic length, *X*, of an interval as
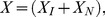
(1)where *X_I_* and *X_N_* are the contributions to the map length of the interfering and the noninterfering crossovers, respectively. In the usual manner ([1], [11]), we define the coefficient of coincidence *C* = (observed coincidences)/(coincidences expected on the hypothesis of independence of crossovers). When map lengths (*X*) are small or interference (1−*C*) is strong, X, expressed in Morgans, is approximately equal to the frequency of recombinants (*R*). For crossover events that are independent of each other within and between pathways, the expected coincidence will then be approximately proportional to the square of the map length 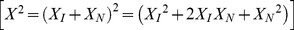. The observed coincidence, however, will depend on the coefficient of coincidence for the interfering crossovers (*S*), so that
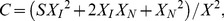
(2)which is more useful when rewritten as



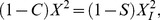
(3a)

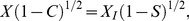
(3b)


Note that Eq. 3a is in terms of interference, (1−*C* and 1−*S*), rather than coincidence, and expresses the obvious – the double crossovers or nonparental ditype tetrads (NPDs) that do not occur in the overall population due to interference (on the left) are simply those that do not occur due to interference in the interfering class of crossovers (on the right). The total map distance (*X*) and the coefficient of coincidence or NPD ratio (*C*), can be calculated from the primary data by conventional methods, but *X_I_* and *S* cannot, in general, be individually evaluated. For short intervals (small *X*), however, we may plausibly assume that *S* is close to zero, as it is in Drosophila (reviewed in, *e.g*., [12]), such that
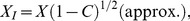
(4)


### Estimating *X_I_* and *X_N_* from Wild-type Tetrad Data

Table 1 illustrates how Eq. 4 allows observed values of *C* to be used for estimating *X_I_* values for six intervals in yeast. The two data sets were collected in zero-growth protocols, which avoid the spurious NPDs that accumulate during growth of diploid cultures. Intervals chosen are “short” by the criterion that *R* and *X* are within 10% of each other. For each of the entries, Table 1 shows *X_I_* values that are modestly less than those of X, with an average *X_I_/X* = 0.87. The result supports the view that, in wild-type meiosis, the noninterfering crossovers are typically a minor fraction of all crossovers (*e.g*., [5]), and is compatible with the view that longer chromosomes (chromosome XV) have a lower relative density of pairing pathway crossovers than do short ones (chromosome III) (*e.g*., [13]). (When *C* refers to two separate intervals, rather than an NPD ratio, *X* and *X_I_* will be the geometric means for the two intervals. The estimates of *X_I_* will be underestimates to the extent that *S* is greater than zero. For *S* less than 0.2, *X_I_* is underestimated by less than 11%. When *S* is greater than zero, *X_N_* is overestimated. Within the framework of Eq. 3, *S* cannot be greater than *C*.).

**Table 1:** *X_I_* and *X_N_* estimated from wild-type yeast tetrad data.

Interval	P:T:N^a^	*R* b	*C* c	*X* d (cM)	*X_I_* e (cM)	*X_N_* f (cM)
URA–LEU	607∶456:5	21.8	0.181	22.8±0.9	20.6±1.0	2.2±1.0
LEU–LYS	496∶569:3	26.9	0.070	27.5±0.9	26.5±0.8	1.0±0.6
LYS–ADE	803∶263:2	12.5	0.229	12.9±0.8	11.3±1.1	1.6±1.2
ADE–HIS	343∶709:16	34.7	0.214	37.7±1.2	33.4±0.9	4.3±1.2
URA3–his4X	855∶356:6	15.1	0.408	16.1±0.9	12.4±1.5	3.7±1.8
HML–his4X	700∶503:10	21.6	0.326	23.2±1.0	19.0±1.2	4.2±1.5

The first four entrie*s* are for chromosome XV [18]; last two entries are for chromosome III [23]. Intervals are those for which *X/R* <1.1.

a Observed PD, TT, NPD tetrads.

b Observed recombinant frequency x 100 = 100(T/2+N)/(P+T+N).

c NPD ratio = N/(N expected in the absence of interference) [3], using the online calculator at http://molbio.uoregon.edu/~fstahl/ncompare2.php.

d Map length in cM (Perkins 1949) [24], using the online calculator at http://molbio.uoregon.edu/~fstahl/compare2.php.

e Calculated from Eq. 4, which assumes that *S* = 0 for these intervals.

f Noninterfering crossovers calculated as *X–X_I_.*

### Searching for True Interference Mutants

Yeast mutants have been identified that reduce interference measured as 1−*C*. Such mutants could result from a reduction in 1−*S*, a reduction in *X_I_* or an increase in *X_N_*., or from some mixture of these. Mutants that affect *S* would be of interest, since they might affect interference in a manner that leads to an understanding of interference *per se*. In a search for “*S* mutants”, it would be helpful to screen out those mutants with altered *C* that are apt to have explanations other than an increase in *S*.

The mismatch repair mutant *mlh1*Δ is an example of a mutant reduced for *X* and increased for *C*. Before seriously entertaining the possibility that the change in *C* is contributed to by a change in *S*, one might wish to exclude the less interesting hypothesis – that *mlh1*Δ is simply a mutant that specifically reduces interfering crossovers. For the condition imposed by the assumptions (above), that we are dealing with short distances, our test for adequacy of the null hypothesis is tantamount to asking whether the change in *C* predicts a reduction in *X_I_* that is indistinguishable from the observed reduction in *X*, while we hold *S* at zero (Eq. 2). In Table 2, which reports interference in terms of NPD ratios, we see that available data are compatible with that view. Thus, these data give us no reason to suspect that *mlh1*Δ is other than a mutant that reduces the frequency of interfering crossovers. (The discrepancy between this conclusion and that offered by Abdullah et al. [14] may be due to the protocol employed by those authors, which permits the accumulation of NPDs in the diploid culture prior to sporulation.).

**Table 2:** Crossing over and interference in *mlh1*Δ mutant.

Interval	Type	P:T:N^a^	C^b^	*X* (cM)^c^	*X*_I_ (cM)^d^	Δ*X* e	Δ*X*_I_^f^
URA–	WT	607∶456:5	0.18	22.8±1.0	20.6±1.0		
LEU	*mlh1*	486∶128:2	0.55	11.4±1.1	7.7±2.9	11.4±1.4	12.9±3.1
ADE–	WT	343∶709:16	0.21	37.7±1.2	33.4±0.9		
HIS	*mlh1*	400∶211:5	0.47	19.6±1.4	14.2±2.3	18.1±1.9	19.2±2.5

Intervals are those for which *X*/*R* <1.1 and N >0 [18].

a Observed PD, TT, NPD tetrads. Sporulation was in a zero-growth protocol, which avoids the NPDs that can arise during growth of a diploid.

b NPD ratio as in Table 1.

d Map length as in Table 1.

d Calculated from Eq. 4.

e Abs. val. [*X* for WT – *X* for *mlh1*].

g Abs. val. [*X_I_* for WT – *X_I_* for *mlh1*].

The mutants *ndj1*Δ and *csm4*Δ, whose phenotypes are identical to each other, increase crossing over while decreasing interference. We may test the simple explanation that *S* is unchanged, remaining at zero, while *X_N_* is increased sufficiently to account for the increase in *X*. These calculations (Table 3) suggest that this simple hypothesis is inadequate for these mutants. However, appropriate increases in both *X_I_* and *X_N_*, with *S* remaining at zero, can account for the observed increase in *C* (Table 3). Thus, again, changes in *S* are not ruled out, but neither can they be claimed without independent evidence regarding *X_I_* or *X_N_*.

**Table 3:** Crossing over and Interference in *csm4*Δ and *ndj*Δ mutants.

Interv	Type	P:T:N	*C*	*X* (cM)	*X_I_* (cM)	*X_N_* (cM)	Δ*X_I_*	Δ*X_N_*
URA–	WT	607∶456:5	0.18	22.8±1.0	20.6±1.0	2.2±1.0		
LEU	Mut.	713∶1045:34	0.35	34.8±1.0	28.2±0.9	6.6±1.3	7.6±1.3	4.5±1.7
LEU–	WT	496∶569:3	0.07	27.5±0.9	26.5±0.8	1.0±0.6		
LYS	Mut.	730∶1041:21	0.23	32.6±0.9	28.6±0.7	3.9±0.9	2.1±1.1	2.9±1.1
LYS–	WT	803∶263:2	0.23	12.9±0.8	11.3±1.1	1.6±1.2		
ADE	Mut.	1170∶613:9	0.30	18.6±0.7	15.5±1.0	3.1±1.2	4.2±1.5	1.5±1.7

Intervals are those for which *X*/R <1.1 and N >0, from Table 1 of Wanat *et al*. (2008) [25]. Data for the three mutant (Mut.) strains *csm4*, *ndj1* and *csm4 ndj1* are combined. Growth conditions and definitions of symbols as in Table 2.

### Estimates of *X_I_* in Wild Type

The presence of both interfering and noninterfering crossovers is widely thought to represent the operations of two pathways for the repair of meiotic double-strand-breaks (reviewed in [15], where they are referred to as “pairing” and “disjunction” pathways, respectively). Mutants *msh4* and *msh5* were, I think, the first identified yeast mutants to decrease both crossing over and interference ([16], [17]) and are closely identified with the pathway leading to interfering crossovers (the “disjunction pathway”). Mutants of *msh4-msh5* are often reported as retaining about 50% of wild type crossing over, encouraging the notion that the pathway leading to interfering crossovers is responsible for about half of all crossing over. However, the results reported here (Table 1), which indicate that interfering crossovers comprise about 90% of the crossover of wild type yeast, are in accord with the 90% value reported [5] for chromosome VII. These views can be reconciled in conventional terms by proposing either (1) that Msh4 and Msh5 are not absolute requirements for the “disjunction pathway” or (2) that there is more than one pathway generating interfering crossovers. Our algebra is independent of those possibilities.

### Mlh1

Argueso et al. [18] (page 1805) remarked that the phenotype of *mlh1*Δ was somewhat mysterious: “A role for *MLH1*-*MLH3* in crossover control is less clear partly because *mlh1*Δ mutants retain crossover interference yet display a decrease in crossing over that is only slightly less severe than that seen in *msh4*Δ and *msh5*Δ mutants.” The mystery is resolved if the interference changes in these mutants reflect primarily the change in the fraction of crossovers of the interfering kind, as suggested above. Then, interference depends on *X_I_* and *X* as 1−*C* = (*X_I_/X*)^2^, which changes rapidly with *X_I_* when a large fraction of the interfering mutants have been lost.

### Ndj1

Getz et al. [19] noted that *msh4*, which reduces interfering crossovers (see above), has no affect on noninterfering crossovers, identified by their failure to repair mismatches that have arisen during DSB repair. Using this mismatch repair feature to identify noninterfering crossovers, Getz et al. [19] concluded that *ndj1* mutants increased the frequency of noninterfering crossovers at the expense of noncrossovers. In their parlance, the pathway leading to noncrossovers and noninterfering crossovers (“pairing pathway”) lacks the ability to repair certain mismatches and is dependent upon Ndj1 to maintain a low crossover frequency. The analysis in Table 3 suggests that the absence of Ndj1 increases interfering crossovers as well as noninterfering ones. In this respect, the phenotype of *ndj1* is reminiscent of that of *rtel-1* in *Caenorhabditis elegans* [20].

## Discussion

The extent to which proposed DSB repair pathways are distinct has been challenged (*e.g*., [21], [22]). Although the algebraic analyses presented above are independent of any such complications, their interpretation in terms of pathways awaits deeper understanding of postulated homeostatic anastomoses.

## Analysis

The analysis is algebra-based.
